# Supplementary material for: Motor unit mechanisms of speed control in mouse locomotion
Source: bioRxiv. 2025 Sep 25:2024.12.29.628022. Preprint. [Version 2] doi: 10.1101/2024.12.29.628022 (PMC12485791; doi:10.1101/2024.12.29.628022)
Supplement: Supplement 1 [file NIHPP2024.12.29.628022v2-supplement-1.pdf]

bioRxiv preprint doi: https://doi.org/10.1101/2024.12.29.628022; this version posted September 25, 2025. The copyright holder for this preprint (which was not certified by peer review) is the author/funder, who has granted bioRxiv a license to display the preprint in perpetuity. It is made available under aCC-BY 4.0 International license.

# Supplemental Figures

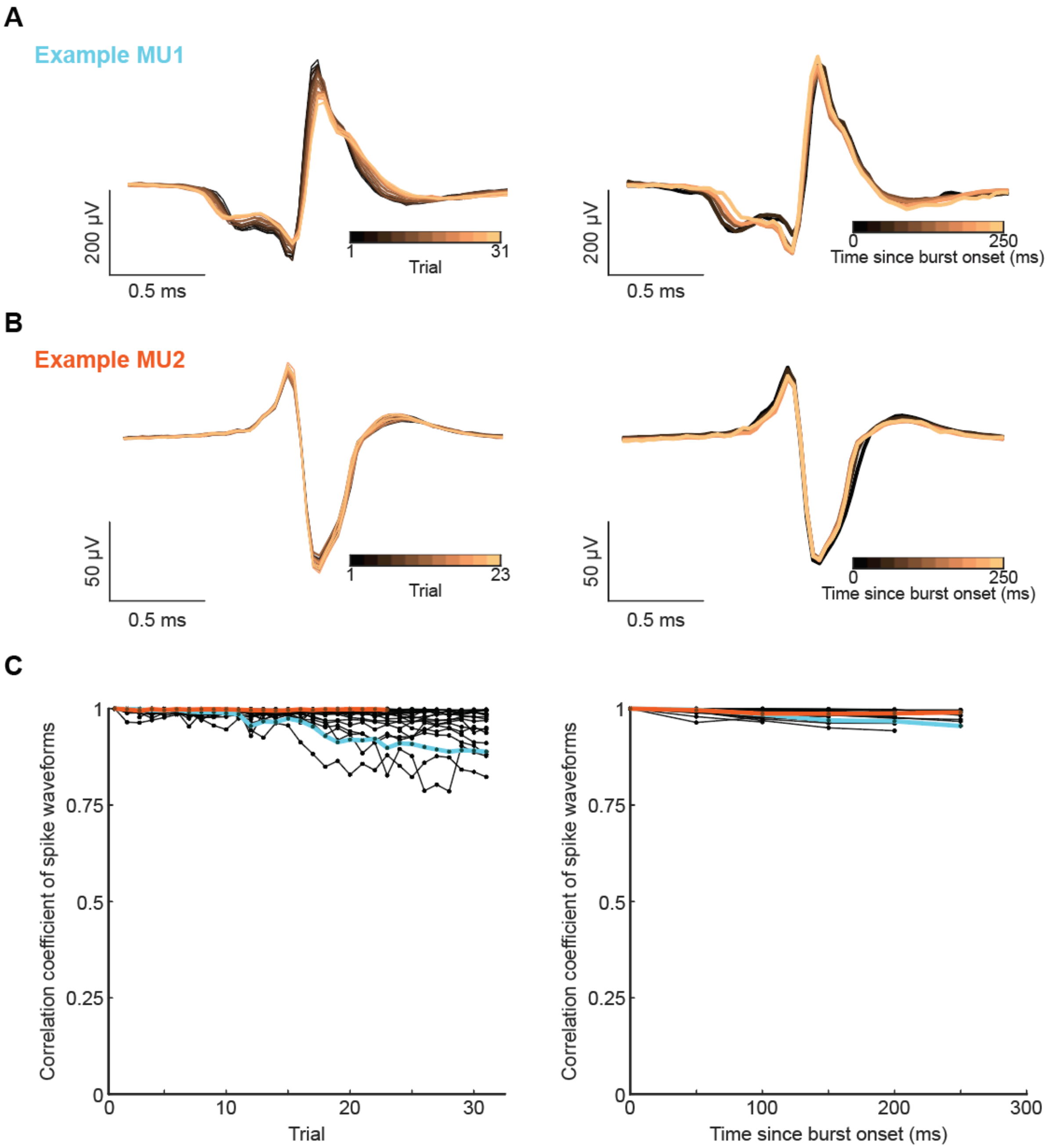


**Figure 1–figure supplement 1.** Isolated motor units had consistent waveforms. **(A,B)** Example motor unit waveforms. (Left) Median waveform calculated from a random subset of strides within each trial. (Right) Median waveform calculated from spikes binned in 50ms increments of the stride. **(C)** (Left) Auto-correlation of each unit's median waveform between the first trial and subsequent trials. (Right) Auto-correlation of each unit's median waveform between the first 50ms of its activity and each subsequent 50ms within the stride.

bioRxiv preprint doi: https://doi.org/10.1101/2024.12.29.628022; this version posted September 25, 2025. The copyright holder for this preprint (which was not certified by peer review) is the author/funder, who has granted bioRxiv a license to display the preprint in perpetuity. It is made available under aCC-BY 4.0 International license.

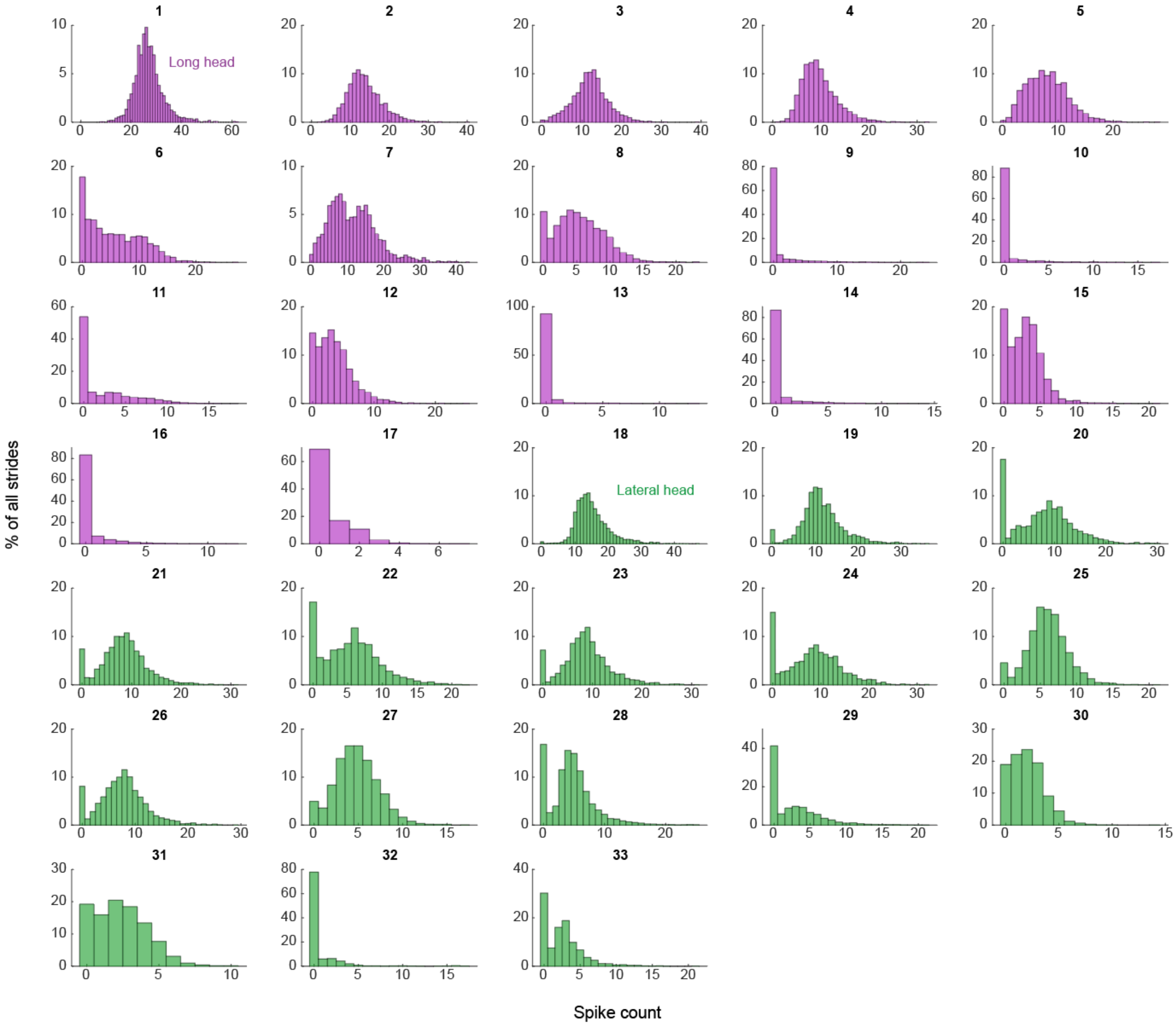


**Figure 2–figure supplement 1.** Empirical observations of spike count distributions for all units. Units are arranged sequentially to match the descending order presented in main text Figure 3. Units 1-17 are in the long head, while units 18-33 are in the lateral head.

bioRxiv preprint doi: https://doi.org/10.1101/2024.12.29.628022; this version posted September 25, 2025. The copyright holder for this preprint (which was not certified by peer review) is the author/funder, who has granted bioRxiv a license to display the preprint in perpetuity. It is made available under aCC-BY 4.0 International license.

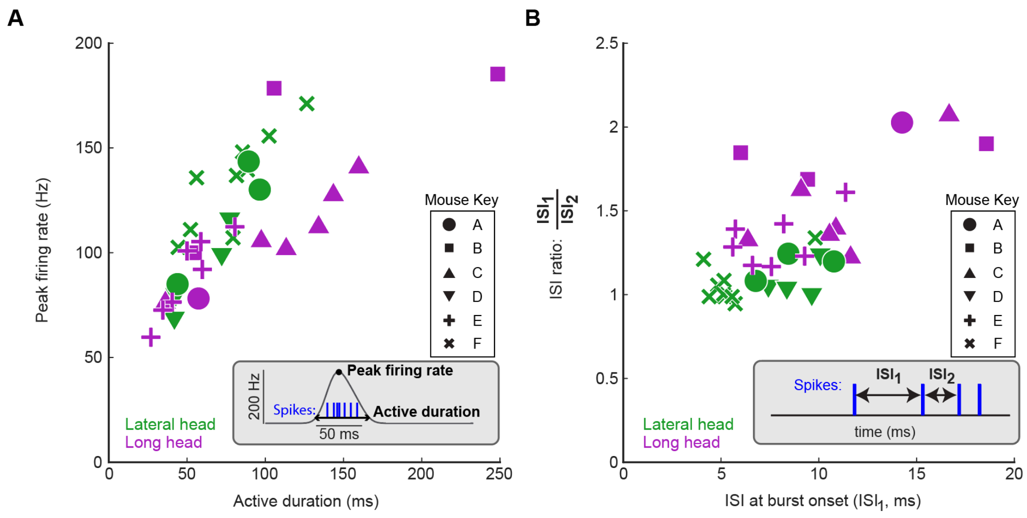


**Figure 4–figure supplement 1.** Symbols denote different animals. All other plotting conventions are the same as in Figure 4 in the main text.

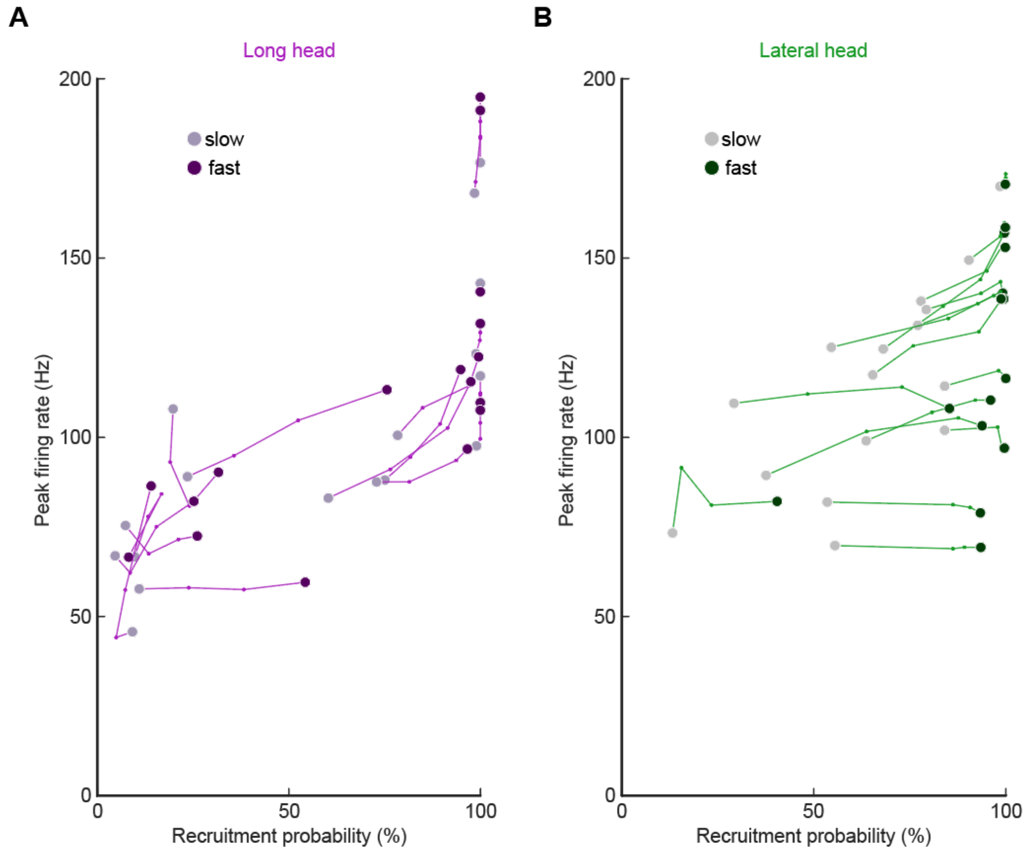


**Figure 5–figure supplement 1.** Altered firing rate and recruitment across walking speed quartiles for all motor units in the long head **(A)** and lateral head **(B)**. Each point reflects the median for the model estimate of each unit across the speeds.

bioRxiv preprint doi: https://doi.org/10.1101/2024.12.29.628022; this version posted September 25, 2025. The copyright holder for this preprint (which was not certified by peer review) is the author/funder, who has granted bioRxiv a license to display the preprint in perpetuity. It is made available under aCC-BY 4.0 International license.

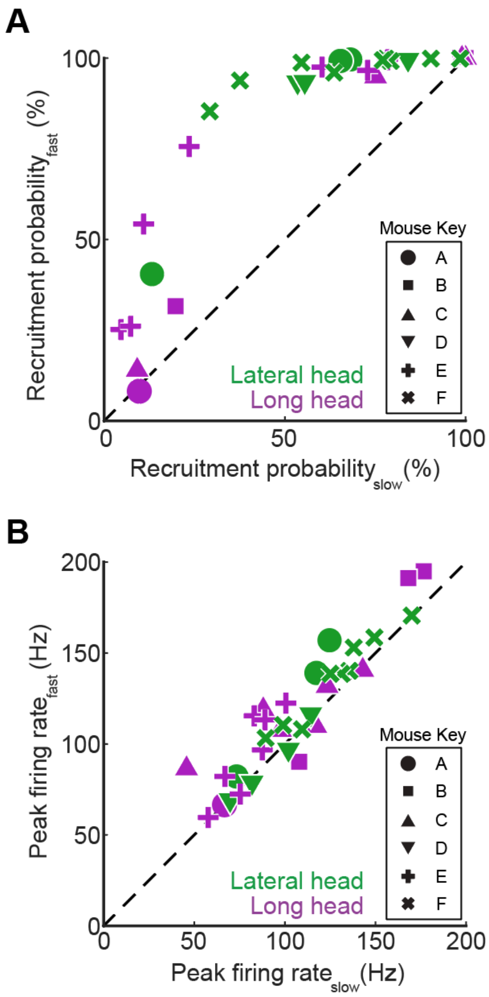


**Figure 5–figure supplement 2.** Symbols denote different animals. All other plotting conventions are the same as in Figure 5 in the main text.

bioRxiv preprint doi: https://doi.org/10.1101/2024.12.29.628022; this version posted September 25, 2025. The copyright holder for this preprint (which was not certified by peer review) is the author/funder, who has granted bioRxiv a license to display the preprint in perpetuity. It is made available under aCC-BY 4.0 International license.

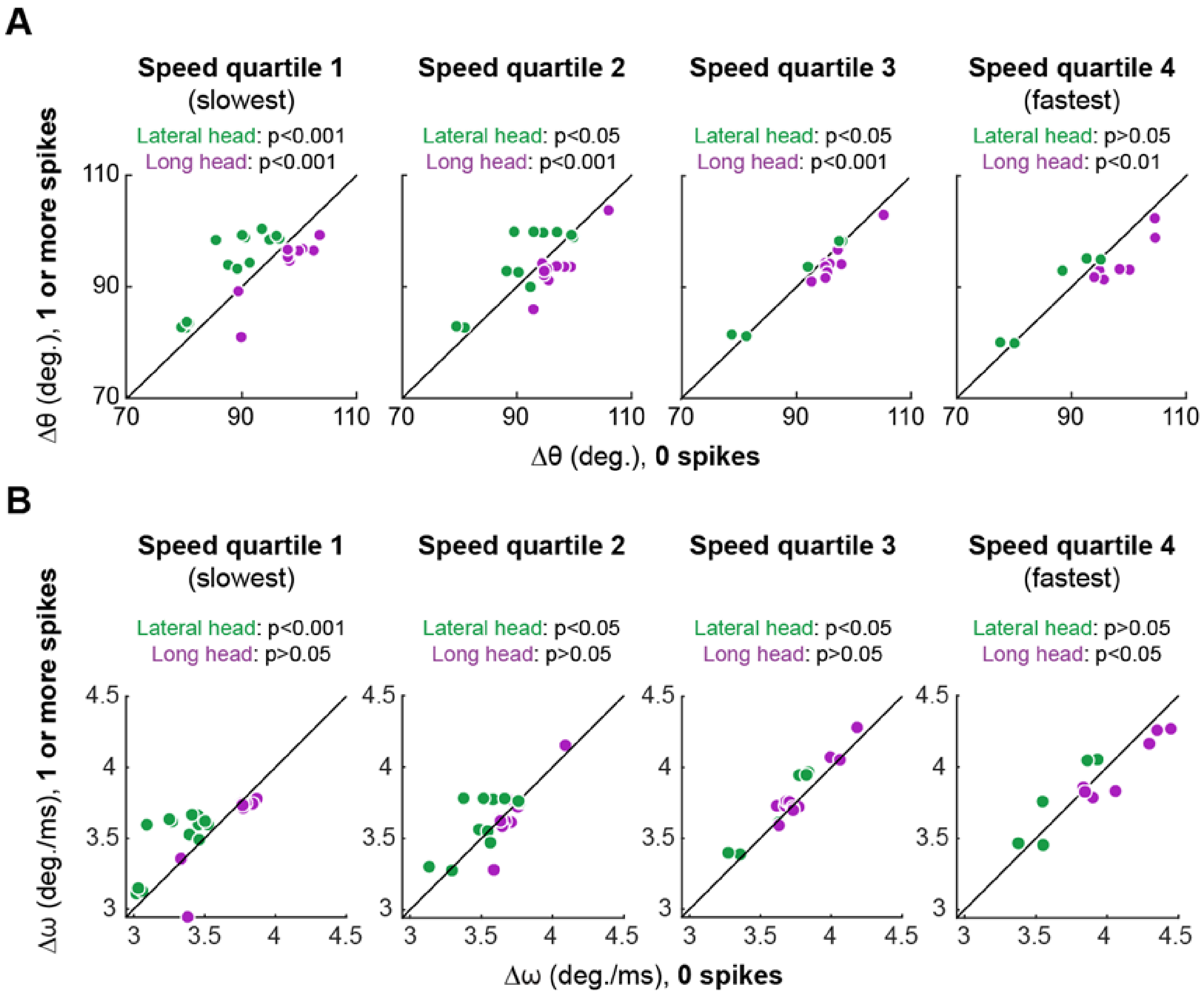


**Figure 6–figure supplement 1.** Plots show the same analysis as main text Figure 6 for each individual speed quartile of **(A)** elbow angle ($\Delta\theta$) and **(B)** elbow velocity ($\Delta\omega$). Speed quartiles 1 and 4 are the slowest and fastest quartiles, respectively, and p-values refer to the results of Wilcoxon signed-rank tests performed separately on data from motor units of the lateral (green) and long (purple) heads of the triceps muscle. Note that most of the muscle-specific differences shown in Figure 6 (C, E) were also present when each of the four quartiles were examined individually for each muscle.

bioRxiv preprint doi: https://doi.org/10.1101/2024.12.29.628022; this version posted September 25, 2025. The copyright holder for this preprint (which was not certified by peer review) is the author/funder, who has granted bioRxiv a license to display the preprint in perpetuity. It is made available under aCC-BY 4.0 International license.

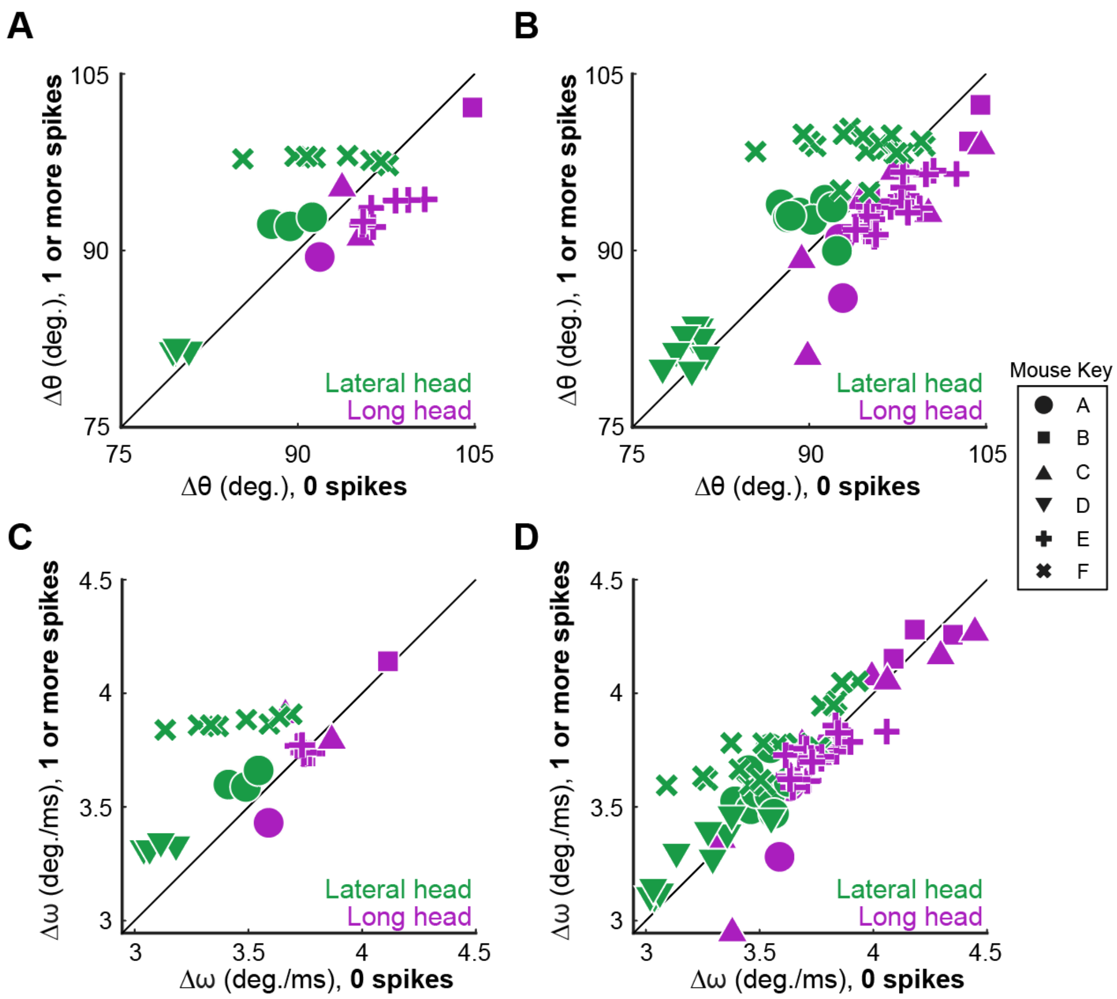


**Figure 6–figure supplement 2.** Symbols denote different animals. All other plotting conventions are the same as in Figure 6 in the main text.
